# Optimization of Enzymatic and Chemical Decellularization of Native Porcine Heart Valves for the Generation of Decellularized Xenografts

**DOI:** 10.3390/ijms25074026

**Published:** 2024-04-04

**Authors:** Monireh Saeid Nia, Lena Maria Floder, Jette Anika Seiler, Thomas Puehler, Nina Sophie Pommert, Rouven Berndt, David Meier, Stephanie L. Sellers, Janarthanan Sathananthan, Xiling Zhang, Mario Hasler, Stanislav N. Gorb, Gregor Warnecke, Georg Lutter

**Affiliations:** 1 Department of Cardiac Surgery, University Hospital Schleswig-Holstein (UKSH), 24105 Kiel, Germany; monireh.saeidnia@uksh.de (M.S.N.); l.floder@gmx.de (L.M.F.); jette-anika.seiler@uksh.de (J.A.S.); ninasophie.pommert@uksh.de (N.S.P.); zhang_xiling@outlook.com (X.Z.); gregor.warnecke@uksh.de (G.W.); 2 DZHK (German Centre for Cardiovascular Research), Partner Site Hamburg/Kiel/Lübeck, 69120 Hamburg, Germany; thomas.puehler@uksh.de; 3 Department of Cardiac Surgery, University Hospital Schleswig-Holstein (UKSH), 23562 Lübeck, Germany; 4 Clinic of Vascular and Endovascular Surgery, University Hospital Schleswig-Holstein (UKSH), 24105 Kiel, Germany; rouven.berndt@uksh.de; 5 Department of Cardiology, Lausanne University Hospital and University of Lausanne, 1015 Lausanne, Switzerland; david.meier1291@gmail.com; 6 Centre for Cardiovascular Innovation, University of British Columbia, Vancouver, BC V5Z 1M9, Canada; ssellers@providencehealth.bc.ca (S.L.S.); jsathananthan@providencehealth.bc.ca (J.S.); 7 Cardiovascular Translational Laboratory, Providence Research & Centre for Heart Lung Innovation, Vancouver, BC V6Z 1Y6, Canada; 8 Centre for Heart Valve Innovation, St. Paul’s Hospital, Vancouver, BC V6Z 1Y6, Canada; 9 Lehrfach Variationsstatistik, Christian-Albrechts-University of Kiel, 24118 Kiel, Germany; hasler@email.uni-kiel.de; 10 Department of Functional Morphology and Biomechanics, Zoological Institute, Christian-Albrechts-University of Kiel, 24118 Kiel, Germany; sgorb@zoologie.uni-kiel.de

**Keywords:** tissue engineering, stent, valved stent, heart valve, valve replacement, decellularization, detergent, enzyme, immunogenicity, DNA residue, extra cellular matrix (ECM) ultrastructure, transcatheter

## Abstract

One of the most important medical interventions for individuals with heart valvular disease is heart valve replacement, which is not without substantial challenges, particularly for pediatric patients. Due to their biological properties and biocompatibility, natural tissue-originated scaffolds derived from human or animal sources are one type of scaffold that is widely used in tissue engineering. However, they are known for their high potential for immunogenicity. Being free of cells and genetic material, decellularized xenografts, consequently, have low immunogenicity and, thus, are expected to be tolerated by the recipient’s immune system. The scaffold ultrastructure and ECM composition can be affected by cell removal agents. Therefore, applying an appropriate method that preserves intact the structure of the ECM plays a critical role in the final result. So far, there has not been an effective decellularization technique that preserves the integrity of the heart valve’s ultrastructure while securing the least amount of genetic material left. This study demonstrates a new protocol with untraceable cells and residual DNA, thereby maximally reducing any chance of immunogenicity. The mechanical and biochemical properties of the ECM resemble those of native heart valves. Results from this study strongly indicate that different critical factors, such as ionic detergent omission, the substitution of Triton X-100 with Tergitol, and using a lower concentration of trypsin and a higher concentration of DNase and RNase, play a significant role in maintaining intact the ultrastructure and function of the ECM.

## 1. Introduction

Heart valve replacement, an essential medical intervention for patients with heart valvular disease, is known for its significant challenges. While mechanical heart valve prostheses require lifelong anticoagulant treatment and have an enhanced risk of thromboembolism [1,2], bioprosthetic valves necessitate replacement at least every 10–15 years [3]. In contrast, tissue-engineered heart valves, resembling native valves in their growth and integration capacity, are promising substitutions, especially for pediatric patients [3,4]. They are facilitated through engineering new and fully functional tissue by the cultivation of the appropriate cells on optimized biological scaffolds [5]. Scaffolds, autologous cells, and growth factors are the main cornerstones of the tissue engineering triad [6]. The scaffold, as the main component, supplies not only the platform for the cells and growth factors [6], but also the mechanical properties and stability, which play an important role in the enhancement of cell proliferation and attachment [5].

Natural tissue-originated scaffolds from human or animal sources, as one type of scaffold widely used in tissue engineering, are favored due to their biocompatibility and biological characteristics [5,7]. Moreover, their scaffold ultrastructure and extracellular matrix (ECM) composition have been shown to positively affect cell differentiation and mitogenesis [4,8]. However, challenges arise when using xenogeneic (animal-derived) scaffolds due to their potential for immunogenicity [4,8]. To mitigate the risk of rejection and inflammation, xenogeneic scaffolds are usually treated with glutaraldehyde [4,9]. Nevertheless, this process may stiffen the fiber network of the ECM and compromise the strengthening function of the spongiosa layer [4,10]. In contrast to glutaraldehyde fixation, tissue decellularization enhances valve integration with a minor risk of inflammation and enables recolonization with host cells while withdrawing tissue antigenicity [11].

Being free of cells and, consequently, also free of genetic materials, decellularized xenografts have low immunogenicity and, therefore, are expected to be tolerated by the recipient’s immune system [12]. Despite decellularization, when the ECM ultrastructure remains intact, it resembles the anatomical and physiological characteristics of native tissue [13]. Therefore, decellularized xenografts are reassuring substitutions for valve replacement with storage potential and, consequently, commercial availability [14].

Xenogenic decellularization is possible through several different chemical, physical, and biological methods, or even the combination of different methods with different strengths and weaknesses [4,15,16]. The scaffold ultrastructure and ECM composition can be affected by cell removal agents. Therefore, applying an appropriate method, and preserving intact the structure of the ECM plays an important role in the end result [4]. However, until now, an optimal decellularization method with the least possible remaining genetic material and the assured intactness of the heart valve ultrastructure has not been developed. In many studies, the decellularization protocols have been optimized to improve ECM preservation and reduce toxicity. Optimization has been tried for by using a new detergent (for example, using Tergitol instead of Triton X-100) [3], a different concentration of the detergent as well as the processing time (for example, sodium deoxycholate, SD) [17], or by adjusting the freeze–thaw temperatures and incubation times [18]. Although these modifications have improved the decellularization outcomes, they have not been able to minimize the DNA content to negligible amounts.

This study introduces a new time-efficient protocol, initially selected by a pilot study (see Section S1.2, DNA quantification, in the Supplementary Materials) in which Triton X-100 was replaced with Tergitol, a new eco-friendly detergent with rapid degradability [3]. Besides, ionic detergents like SDS were avoided due to their significant disruption of protein structures, and their removal of growth factors and glycosaminoglycans (GAGs) [4,5]. and a lower trypsin concentration and a higher concentration of DNase and RNase were used in this protocol.

Our results using this protocol clearly show untraceable cells and DNA remaining, mitigating the risk of immunogenicity, as well as demonstrating the comparable mechanical and biochemical properties of the ECM to native heart valves. Therefore, this protocol is a promising approach for providing a biocompatible alternative to mechanical heart valve prostheses with minimum immunogenic responses and native valve-resembling biomechanical properties, which are crucial issues in heart valve replacements. 

## 2. Results

After conducting a pilot study to analyze 23 decellularization protocols (the main differences among all the evaluated protocols in the pilot study are highlighted in Table S1 in the Supplementary Materials), which differed in the types of detergents, concentrations, and also the treatment durations used, the residual DNA after decellularization by each protocol was analyzed and compared (the results are presented in Figure S2 in Section S1.2, DNA quantification, in the Supplementary Materials). Among these decellularization protocols, the one with the best outcome, with the lowest DNA residual after a shorter time and intact visual appearance, was selected for further investigations of its effects on the morphological and mechanical properties, as well as the ultrastructure, of the ECM. The following section presents the key findings and outcomes of the conducted research, highlighting the significant results and their implications. The evaluation of the porcine aortic and pulmonary heart valves was conducted for three different states: native, decellularized, and reseeded. 

### 2.1. Visual Appearance 

**Figure 1:**
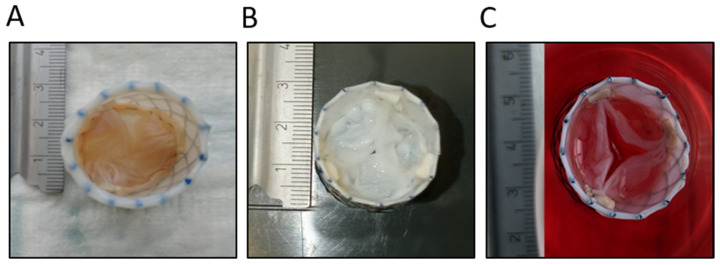
Representative images of the native aortic valve (**A**), the decellularized aortic valve (**B**), and the recellularized aortic valve (**C**). All the images have an aortic view.

The inspection of both the native porcine aortic and pulmonary valves showed a delicate-pink-to-fading-light-brown coloring overall (Figure 1A). After decellularization with the newly optimized method, the leaflets presented with a shinier surface, while being undamaged and still preserving their fine structure (Figure 1B). The valves appeared off-white in total. Upon careful examination, it was determined that the decellularized valves exhibited an absence of any shrinkage or swelling, thereby validating the efficiency of the decellularization process for ensuring the preservation of the valves’ structural integrity. After recellularization, both the aortic and pulmonary valves showed a light-pink color. The structure of the leaflets was retained and there was no visible damage. Furthermore, the valves were neither shrunken nor swollen in size. The texture of the recellularized cardiac valves remained flexible and absent of brittleness (Figure 1C).

### 2.2. DNA Quantification

**Figure 2:**
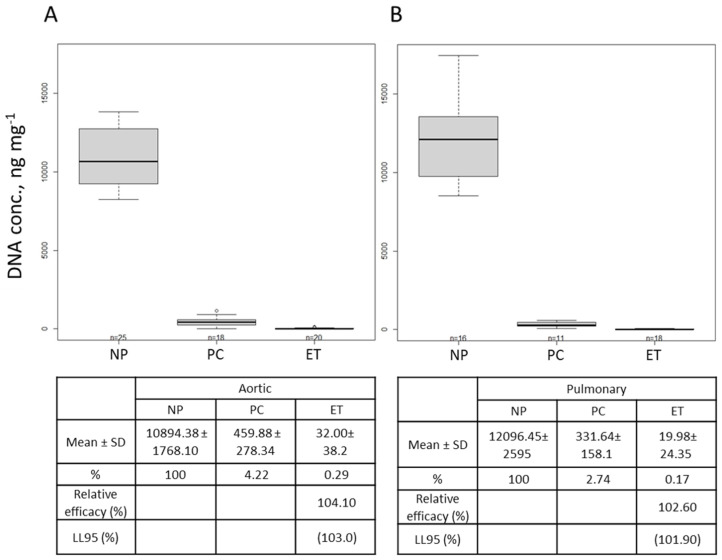
Absolute (boxplots) and relative (tables) quantification of the DNA content of the untreated native and decellularized aortic (**A**) and pulmonary valves (**B**). In the tables, the DNA concentrations are presented as the mean ± SD and percentages, the relative efficacy (%) of the experimental treatment (ET) compared to the ‘home decellularization method’ (positive control, PC), and the lower limit of the corresponding 95% confidence interval (LL95).

After decellularization, the DNA was isolated and quantified (see Section 4.4 DNA Isolation and Quantification). The results from the DNA content of the tissues showed a reduction in DNA in comparison to the native porcine leaflets (NP). In comparison to the home decellularization method (positive control, PC), the results from the modified decellularization method (experimental treatment, ET) showed a decrease in the DNA content to nearly zero for both the aortic and pulmonary valves (Figure 2A,B). The relative efficacy of the ET when compared to the PC was determined to be about 104% and 103% in the case of the aortic and pulmonary valves, respectively. The lower limits of the corresponding 95% confidence intervals (103% and 102%) indicated a significant performance improvement (Figure 2A,B). 

### 2.3. Biomechanical Test

**Figure 3:**
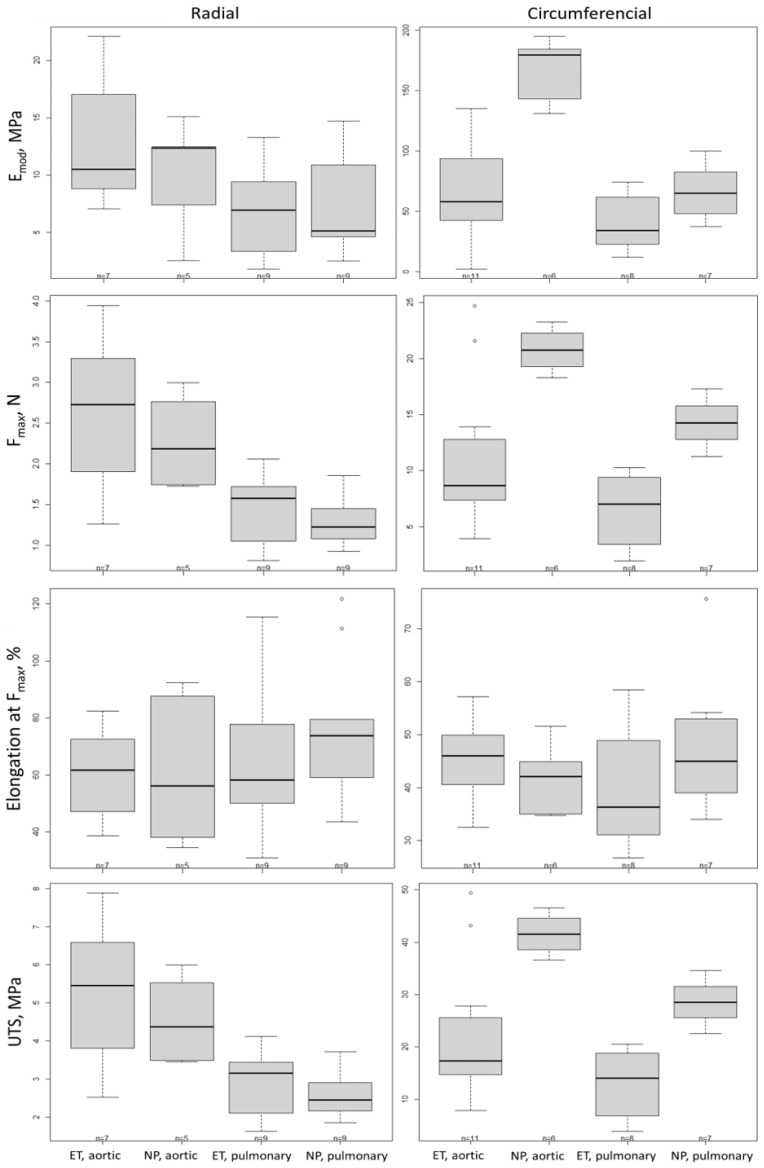
Uniaxial tensile mechanical properties shown as boxplots for E_mod_, F_max_, elongation at F_max_, and UTS of native aortic and pulmonary xenografts decellularized by experimental treatment (ET) in comparison to aortic and pulmonary native porcine (NP) in both radial and circumferential directions.

The mechanical properties of the decellularized xenografts with the experimental treatment (ET), such as Young’s modulus (E_mod_), the maximum stress at break (F_max_), the mean strain at break (elongation at F_max_), and ultimate tensile strength (UTS), were determined and compared to those of native porcine (NP) aortic and pulmonary valves through a behavior analysis under uniaxial loading, in both the radial and circumferential directions (Figure 3). The results from this analysis, indicating the potential equivalence of the ET and NP, are presented in Table 1. For example, the results from the analysis of E_mod_ in the radial direction indicate that the ET and NP (aortic) differ by 3.06 MPa (Table 1). If one accepts the equivalence ranges (±8.7 MPa, Table 1), the ET and NP are significantly equal regarding E_mod_ in the radial direction. Therefore, from the authors’ perspective, the biomechanical analysis shows that the ET and NC are significantly equal in their mechanical properties in the radial direction (Table 1). However, the results from the analysis in the circumferential direction show that the ET and NP are significantly equal (from the authors’ perspective) only regarding the elongation at F_max_ (for both the aortic and pulmonary valves) and E_mod_ (for the pulmonary valves).

### 2.4. Histology and Immunohistochemistry Analysis

**Figure 4:**
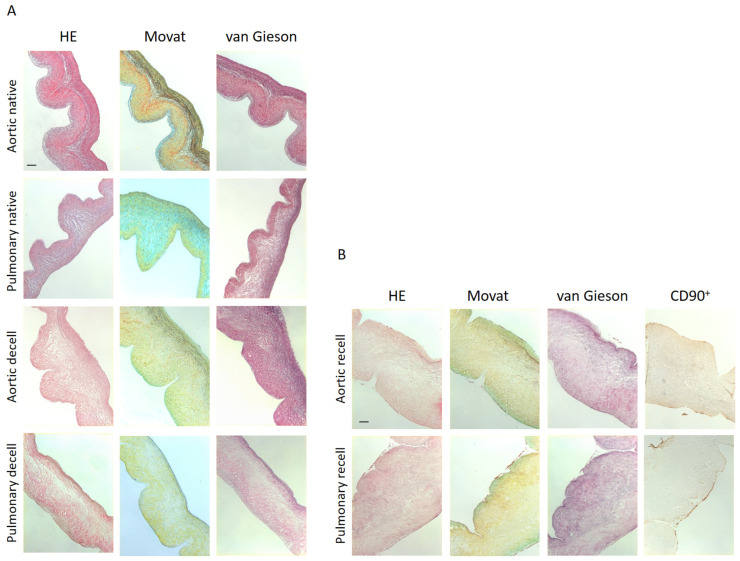
Representative images of aortic and pulmonary leaflet tissues before decellularization (native) and after decellularization (decell) (**A**), and after recellularization (recell) (**B**), and stained with HE, Movat, van Gieson, and CD90^+^. The scale bar indicates 100 µm (**A**,**B**). After Movat pentachrome, an intact ECM consisting of nuclei and elastic fibers can be distinguished in black, loose connective tissue with collagen fibers in yellow, cytoplasm and proteoglycans in red, and glycosaminoglycans in greenish blue.

The untreated aortic and pulmonary valves showed distinct layers with smooth connections and minimal disruptions (Figure 4A). The Movat pentachrome staining validated this with an evident presentation of an intact ECM, consisting of the nuclei and elastic fibers in black, the loose connective tissue with collagen fibers in yellow, the cytoplasm in red, as well as the proteoglycans and glycosaminoglycans in greenish blue (Figure 4A). After undergoing our refined decellularization protocol, the ventricularis, the spongiosa, as well as the fibrosa layer were well preserved and similar to the native one, resembling the native tissue in terms of its structure, mechanical properties, and biofunctionality (Figure 4A). The heart leaflet and valve appeared structurally intact and free from cellular components. All the nuclei were successfully removed. The edges of the leaflet were unscathed; therefore, the leaflet also maintained its original shape and dimension (Figure 4A). In the Movat pentachrome staining, the levels of glycosaminoglycans appeared diminished, while the collagen remained intact. In this staining, for the case of the native aortic sample, the reddish-yellow coloring of the collagen areas was due to the lower incubation time in the yellow stain solution. The Movat pentachrome and Elastica van Gieson staining additionally revealed intact elastin fibers. A similar image presented itself in the HE as well as in the Elastica van Gieson and CD90^+^ stainings (Figure 4A). In the HE staining, as an overview staining, the nuclei along with the cytoplasm of the fibroblast cells were no longer visible. However, the collagen and cytoplasmic components were stained with Eosin. The structure of the extracellular matrix remained unchanged and there was no noticeable swelling throughout the leaflet. The edges of the leaflet were smooth and nondamaged (Figure 4A). Regarding the Elastica staining, the elastic fibers were stained dark purple by Weigert’s resorcin–fuchsin solution and showed no disruption of the elastic fibers, as well as intact edges (Figure 4A). An immunostaining confirmed the cells seeded on the decellularized heart valve leaflets to be CD90^+^ after recellularization (Figure 4B). The cells colonized the surface of the tissue in a cohesive manner, proving the non-toxicity of the tissue after treatment with the newly developed decellularization protocol (Figure 4B). However, there were no signs of ingrowth after 21 days of seeding. The structure of the ECM after decellularization and recellularization was comparable (Figure 4A,B).

### 2.5. Scanning Electron Microscopy and ECM Topography

**Figure 5:**
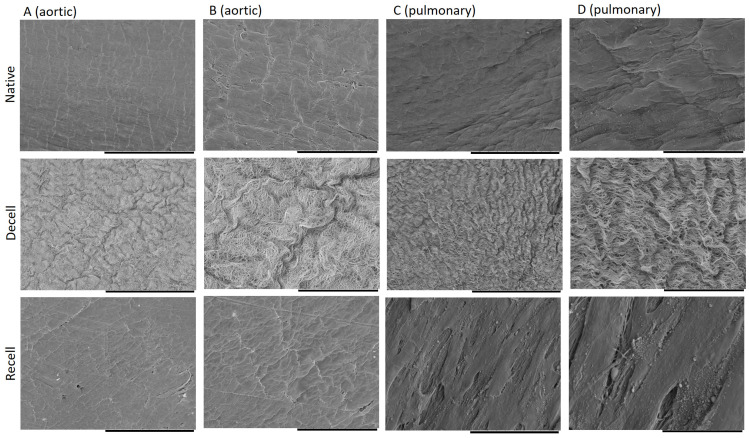
Representative scanning electron microscopic images of aortic (**A**,**B**) and pulmonary (**C**,**D**) leaflet tissues before decellularization (native), after decellularization (decell), and after recellularization (recell). The scale bar indicates 100 µm (**A**,**C**) and 30 µm (**B**,**D**), respectively.

The decellularized heart valves presented completely cell-free surfaces with undulated and well-structured fiber masses (Figure 5A–D). The nuclear components were entirely missing and there was no reduction in thickness visible in comparison to the native valves. For recell purposes, human induced-pluripotent stem cells-derived MSCs (hMSCs, described before by [19]) were used. The recellularized compound mostly showed fusiform cell structures with an extracellular matrix in-between, but also confluent cell layers on top. In comparison to the native heart valve, there was increased microvillus formation (Figure 5A–D).

### 2.6. Morphological Results of the Valved Stent

**Figure 6:**
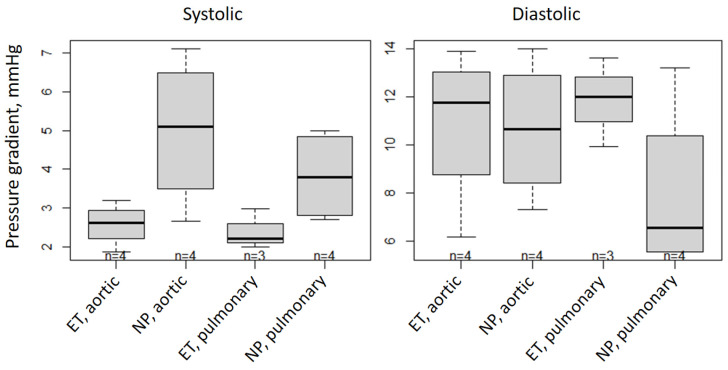
Systolic and diastolic pressure (mmHg) gradients shown as boxplots for native aortic and pulmonary xenografts decellularized by experimental treatment (ET) in comparison to aortic and pulmonary native porcine (NP).

**Figure 7:**
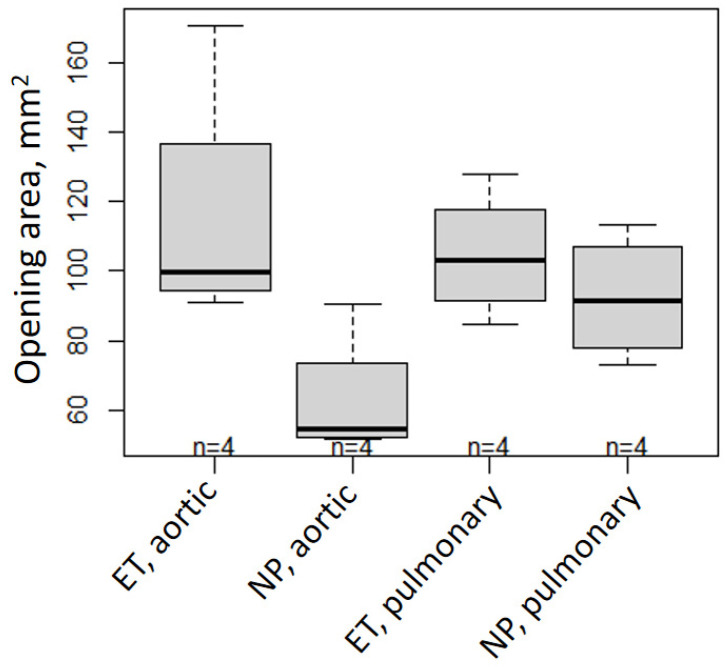
Valve opening areas (mm^2^) shown as boxplots for native aortic and pulmonary xenografts decellularized by experimental treatment (ET) in comparison to aortic and pulmonary native porcine (NP).

Table 2 presents the pressure gradients for the aortic and pulmonary valves during systole and diastole (see also Figure 6). For systole, lower pressure gradients are preferred, and for diastole, higher pressure gradients are preferred. While in systole, the aortic gradient difference is not significantly equal in the ET and NP, with a mean difference of −2.41 mmHg (which means an even lower systolic pressure gradient in the ET than in the NP); in the other cases, the ET and NP samples are significantly equal. Our results show that the aortic valve opening area at 60 bpm (Figure 7) is not significantly equal in the ET and NP, with a mean difference of 52.4 mm^2^. However, the pulmonary valve opening areas are equal in both ET and NP (Table 3).

## 3. Discussion

Heart valves encompass a thin ventricularis layer containing elastic fibers, the spongiosa layer with collagen fibers and glycosaminoglycans in the middle, and the fibrosa with dense collagen fiber bundles [20,21]. Our newly developed protocol, presented in this study, shows significant differences not only macroscopically, but also under a scanning electron microscope, with a fully intact endothelial cell layer surrounding the fibrosa and ventricularis (Figure 5). Similarly, the histological analysis clearly shows that all three layers are well preserved, resembling the native tissue in terms of its structure, mechanical properties, and biofunctionality (Figure 4). Moreover, the biomechanical analysis (Figure 3 and Table 1) indicates that xenografts decellularized by the experimental treatment (ET) presented in this study have equal mechanical properties as the native ones when measured in the radial direction (Table 1). However, the different mechanical properties of the decellularized xenograft compared to the native control in the circumferential direction can putatively be explained by the increased fiber mobility, due to the less bounded collagen network after decellularization [22]. Since there is a positive correlation between the collagen cross-link concentration and the modulus of elasticity in human aortic valves, the collagen cross-links are suggested as playing an important role in mechanical behavior determination [23]. Moreover, since the orientation and alignment of collagen fibers is a critical factor in mechanical damage, and collagen and elastin fibers can also act as calcium binding sites, collagen fibers that are misaligned with the in vivo principal strain direction also have the potential to exacerbate calcification [24]. Therefore, the damage caused by trypsin and the Tergitol ECM components, especially collagen disruption, is possibly one of the other factors explaining the results of the biomechanical analysis in the circumferential direction [4,25,26]. The results from this study highly suggest that different critical factors, such as SDS omission, Triton X-100 replacement with Tergitol, a lower trypsin concentration, and a higher concentration of DNase and RNase, are putatively mainly involved in preserving intact the ultrastructure and function of the ECM. 

Moreover, in this protocol, in comparison to the protocols described by others [16,27], the duration of treatment with a non-ionic detergent, Tergitol, was reduced from 51 or 120 h, respectively, to only 36 h. The detergent exposure time has been shown to affect the ECM structure. It was reported by He et al. [28] that a shorter SDS treatment time was accompanied by the improved retention of a whole kidney’s ECM structure and function. In another research study by Poornejad et al. [29], reducing the SDS exposure time from 36 to 5 h was reported to preserve the microstructure of a whole porcine kidney. Additionally, Xu et al. [30] showed that shortened SDS and Triton X-100 exposure times improved the biomechanical properties of decellularized whole porcine entheses. Therefore, the Tergitol treatment time reduction in our protocol could presumably be considered as another factor involved in maintaining the integrity and functionality of the decellularized xenograft. However, to know about the effect of the Tergitol treatment time on the ECM integrity, more experiments and further analysis need to be conducted. Apart from the potential effect of the Tergitol treatment time on the ECM integrity and structure, the shortened exposure time results in a more time-efficient protocol. 

The decellularization method’s efficiency must be evaluated concerning its cell removal effectiveness while preserving the microstructure of the ECM [4]. This evaluation consists of a variety of analyses, including DNA quantification, mechanical analysis, visual surface evaluation by a SEM, and histological and immunohistochemical analyses [4,8].

Ionic detergents, such as sodium dodecyl sulfate (SDS), are known to effectively remove cellular material, solubilize cell membranes, and detach DNA from proteins [4,8]. However, due to their significant disruption of protein structures, as well as the removal of glycosaminoglycans (GAGs) and growth factors, ionic detergents cause the loss of matrix components and their functionality [4,5,31,32]. SDS has also been reported to disrupt collagen integrity and cause damage to the ECM structure [32,33], and reduce the mechanical strength of the decellularized ECM [32]. Hence, in our optimized protocol, we deliberately avoided the use of SDS to circumvent the above-mentioned side effects, due to the difficulty of thoroughly washing out any residue of SDS. The latter is caused by its interactions with ECM proteins which, in turn, can induce cytotoxicity, especially in repopulated cells [32,34].

In addition, in decellularization, Triton X-100, a non-ionic detergent, is commonly used to remove remaining ionic detergent [35], and also due to its efficient removal of cellular content by disrupting lipid–lipid and protein–lipid bonds [36]. Since maintaining GAGs and lipids is crucial to preserve the ECM structure and integrity [5], Triton X-100 is replaced here by Tergitol, which is a mild and more environmentally friendly detergent [37]. Our results show clearly that while the structure of the ECM and its protein content stayed intact and preserved, the nuclear component was efficiently removed by using Tergitol (Figure 4). Our results are similar to the results from Tondato et al. [37], where they showed that Tergitol can be a reliable replacement for Triton X-100.

Enzymatic agents, such as trypsin, DNase, and RNase, are also being used in valve decellularization to facilitate cell removal and biomolecule breakdown [38]. A serine protease, trypsin, is known to break protein peptides hydrolytically and, therefore, is commonly used to digest proteins in different decellularization protocols [28,35]. Nevertheless, it has been suggested [25,31] to avoid or reduce the usage of trypsin, as it has been reported to reduce tensile strength, flexural stiffness, and elastin content, as well as causing collagen disruption, and the breaking and deranging of elastin fibers [4,25,26]. The use of Triton X-100 in combination with trypsin has shown complete cell removal from the conduit in rat aortic valves, albeit at the expense of elastin and GAG loss and collagen fiber disruption [39]. Similarly, Yang et al. [25] reported that a combination of Triton X-100 with sodium–deoxycholate led to broken elastin fibers, but that the fiber arrangement was still detectable. Results by Haupt et al. [4] showed that cells on porcine pulmonary valves treated with trypsin were still not completely removed. Moreover, the collagen-rich fibrosa was probably degraded, due to the collagenolytic activity of trypsin against collagen type I [4,40]. Therefore, in this study, besides replacing Triton X-100 with Tergitol, we also used a reduced amount of trypsin, from 0.05% to 0.03%, in comparison to the protocol described in our previous publication [4]. 

Since residual DNA triggers immunological responses in the receiver’s body, a DNA content analysis is an indispensable requisite for the evaluation of decellularization protocol efficiency [3]. Therefore, an ideal decellularization method needs to diminish the risk of animal valve immunogenicity while preserving intact the structure of the ECM and its functionality. In the protocol described in this paper, higher concentrations of DNase and RNase were used in comparison to the protocols described by others [4,16,27], which lessened the DNA content of the valve leaflets effectively and nearly to a negligible amount (Figure 2). Although a high SDS concentration has been reported to have a critical role in decellularization and in lowering DNA residues [32], the absence of SDS in our protocol suggests that SDS can easily be replaced by DNase and RNase for its effect on removing DNA. In this way, the SDS interactions with the ECM [19,21], its collagen integrity disruption, and ECM structure damages [32,33] have been prevented (Figure 4 and Figure 5).

The main goal of decellularization is to minimize the amount of residual genetic material while ensuring that the scaffold ultrastructure of the heart valve is intact. In the morphological observations after decellularization, we did not observe stent fracture or valve tearing (Figure 1). In the mechanical performance tests, there was no significant difference between the ET and NP in terms of either the gradient or opening area (Figure 6 and Figure 7). Moreover, during systole, the aortic gradient difference and the opening area of the ET were superior to those of the NP. Compared to the pulmonary valve, the aortic annulus had more fibrous tissue. This tissue is difficult to remove completely without damaging the leaflets, leading to the possibility of strained leaflets during the fabrication of a native aortic valve stent and causing outflow track stenosis. After decellularization, these fibrous structures may be impaired, but still have the mechanical properties to keep the valve working. The valve leaflets will loosen for this reason and return to normal operation.

## 4. Materials and Methods

### 4.1. Valve Stent Preparation and Methods for Decellularization Process

In compliance with ethical guidelines and local regulations, fresh and intact porcine hearts were provided by a local slaughterhouse. Upon receiving the hearts, they were stored in a freezer until usage to maintain their freshness and integrity. At the outset, both the pulmonary and aortic valves were detached and cleared from the surrounding tissues, like fat and the myocardium. For recell purposes, and also to test the valves on the valve tester, the valves were additionally sutured into NiTinol stents. Before treatment, the valves received an antibiotic pre-treatment (Amphotericin B, Gibco, Scotland, UK; 1% in PBS and Pen Strep, Gibco; 1% in PBS) to remove and prevent any potential microbial contamination during the decellularization process.

### 4.2. Home Decellularization Method 

As described before [4,27], the decellularization started with hypotonic shock by the incubation of the valve in double-distilled water at 4 °C for 24 h. Cell removal was initiated by using 0.1 M sodium hydroxide at RT for 2 h. Afterward, the leaflets were treated with detergents, including 1% SDS, 1% Triton X-100, 1% sodium deoxycholate, and 0.2% EDTA in 50 mM TRIS, pH 7.5, for 6 days with the solution changed every 2 days. To remove nucleic acids, deoxyribonuclease and ribonuclease (720 mU/mL) were used for 4 days at 37 °C, with a fresh solution every 2 days. In the end, the leaflets were sterilized with 0.1% peracetic acid in 1× DPBS, pH 7.5, for 1 h at 22 °C. To avoid or reduce the bioburden and detergent remains, each step was followed by an appropriate washing with 70% ethanol, distilled water, or DSPBS [4,27]. This protocol was used as the positive control (PC).

### 4.3. Modified Decellularization Method

For the newly developed protocol or so-called experimental treatment (ET), a chemical and an enzymatic approach were combined as well. As a new approach, Tergitol was used to maintain the structural and functional integrity of the ECM instead of Triton X-100. In short, the decellularization process was performed on a platform shaker, starting with three rinsing circles with distilled water (5 min, 10 min, and 10 min, respectively), after preparation of the valve leaflets or valved stent to remove of all excess blood and debris. Thereafter, the valves were treated with 0.02% sodium azide at room temperature for five hours to prevent microbial growth and to preserve the leaflets. The solution was changed after 2.5 h. The treatment was resumed with two rinses with distilled water for 10 min each. The samples were incubated for an hour in a 0.05 M NaOH solution on a shaker. Afterward, the tissue was rinsed three times (two times for 5 min and one time for 10 min) with distilled water. The leaflets or valved stent were treated overnight at 4 °C with 1% Tergitol dissolved in 5 mM Tris-HCl buffer solution, which was used to increase the permeability of the cell membranes [41]. 

The following day, 1 µM PMSF was added to the solution at 4 °C, and placed on the shaker for another 6 h. Then, the valves were rinsed three times (one time for 5 min and two times for 10 min) in distilled water to remove the detergents. Thereafter, the valves were treated with 70% EtOH at room temperature for 30 min, followed by another three rinses (each for 5 min) in distilled water. An amount of 625 U DNase and 617.5 U RNase with 0.5 mmol CaCl_2_-Dihydrate were added to 5 mM MgCl_2_ in 0.02% sodium azide in DPBS. The samples were incubated in this solution at 37 °C overnight under agitation.

The next day, the valves were treated with 5 mM MgCl_2_ in 0.02% sodium azide in DPBS solution with 0.035 g trypsin and 2 mmol CaCl_2_-Dihydrate for 2 h at 37 °C. To remove any solvent residue, the valves were rinsed two times in DPBS (5 min each) and four times (two times for 5 min and two times for 10 min) in distilled water at room temperature. Afterward, the samples were treated in 1% Tergitol in 5 mM Tris-HCl overnight at room temperature.

On the last day, 200 mmol sodium chloride was added to the solution and the incubation continued for six hours at room temperature. The samples were rinsed four times (two times for 5 min and two times for 10 min) with DPBS, and then were kept in DPBS at 4 °C for further analysis.

### 4.4. DNA Isolation and Quantification

The applicable leaflets were halved and dried overnight at 55 °C. The next day, after weighing, the dried leaflets were treated with a lysis buffer consisting of SDS, EDTA, Tris-HCl, saturated sodium chloride, distilled water, and proteinase K for 3 h at 55 °C. The samples were then cooled at 4 °C for 10 min, followed by centrifugation (10 min at 13.2 rpm at 4 °C) to separate the cellular debris and proteins from the DNA. Then, the DNA-containing supernatant was treated with 100% Isopropanol for 15 min at −20 °C. After centrifugation for three minutes at 13.2 rpm at 4 °C, the pellets were washed with 70% Isopropanol and 80% EtOH, respectively. Finally, the pellets were dissolved in 100 µL DNase-free water and the DNA concentration was measured by spectrophotometry with a NanoDrop 2000C (Thermo Scientific, Waltham, MA, USA). The DNA concentration was calculated as ng per mg dry weight of the leaflets.

### 4.5. Histology and Immunohistochemistry

The leaflets were fixed in 4% Formalin (Huberlab, Aesch, Switzerland) for four hours before being embedded in paraffin (Merck, Darmstadt, Germany). For the overall tissue analysis, 5 µm sections of the samples were stained with hematoxylin and eosin for a general overview, as well as Movat’s pentachrome and Elastica van Gieson staining to visualize the multiple layers of heart valve leaflets. In addition, a CD90 antibody staining was performed to confirm recolonization of the decellularized tissue. 

Before staining, the sections underwent a deparaffination and rehydration process starting with Roticlear (Roth, Germany), followed by a descending ethanol (Huberlab, Switzerland) series, and a final step in ddH_2_O. After staining, the same steps were taken in reverse to dehydrate the samples. All the sections were mounted in Eukitt (Merck, Rahway, NJ, USA).

#### 4.5.1. HE Staining

After deparaffination and dehydration, the slides were stained with Mayer‘s hemalum solution (Merck, USA) for three minutes and were differentiated under running tap water. Afterward, they were stained for 3 min with 0.5% Eosin G solution (Roth, Germany) enriched with glacial acetic acid (Roth, Germany). 

#### 4.5.2. Movat Pentachrome 

For Movat’s pentachrome, a respective Strain Kit (ScyTek Laboratories, Logan, UT, USA) was used. The sections were deparaffinized and hydrated with distilled water. Briefly, the sections were successively processed in a working elastic solution (consisting of hematoxylin 5%, ferric chloride 10%, and Lugol’s iodine solution), a ferric chloride differentiating solution (2%—twenty dips), a sodium thiosulfate solution (5% for one minute), an alcian blue solution (pH 2.5—thirty minutes), a Bieberich scarlet acid fuchsin solution (two minutes), a phosphotungstic acid solution (5% for fourteen minutes in total), an acetic acid solution (1% for three minutes), and a yellow stain solution (twenty minutes). 

#### 4.5.3. Elastica Van Gieson

For the Elastica van Gieson staining, the slides were dewaxed and rehydrated with ethanol 80%. The main components of the Elastica staining were a resorcin–fuchsin solution (Roth, Germany), Weigert’s iron hematoxyline (Roth, Germany), bluing under running tap water, and staining with van Gieson solution (Roth, Germany). 

#### 4.5.4. Immunohistochemistry: CD90^+^ Antibody Staining

The immunohistochemistry was performed for human-induced pluripotent stem cells-derived MSCs (hMSCs, described by Lutter et al. in [19]). Antigen retrieval was attained through boiling the slides in citrate buffer (10 mM citric acid monohydrate (Merck, USA), 0.05% Tween (AppliChem, Darmstadt, Germany) in ddH_2_O), pH 6. The slides were cooled down for 20 min, before blocking the endogenous peroxidase activity by using a 3% H_2_O_2_ solution (Merck, USA). The sections were further blocked with goat serum (diluted to 10% with PBS, Invitrogen, Waltham, MA, USA) and incubated with a rabbit antihuman CD90 monoclonal antibody (1:150 dilution, Abcam, Cambridge, UK) overnight. Immunodetection was performed by using Histofine^®^ Simple Stain MAX PO (Multi) (Nichirei Biosciences Inc., Tokyo, Japan) according to the manufacturer’s procedures. Conclusively, the slides were counter-stained with Mayer’s hemalum solution (1:5 dilution). The primary antibody was not included in the negative controls.

### 4.6. Scanning Electron Microscopy (SEM)

Micrographs were taken by a scanning electron microscopy (Hitachi TM3000, Hitachi High-Technologies Corporation, Tokyo, Japan). The sample preparation was performed as explained before by Haupt et al. (2018) [4]. After fixation with glutaraldehyde 2.5%, the leaflets were washed with DPBS to remove excess water, and the leaflets were dehydrated with ethanol at ascending concentrations from 30% to 100%. To dry the samples, the protocol for critical-point drying, described by Scherge and Gorb [42], was used. A 10 nm thin gold/palladium layer was sputtered on the samples to enhance conductivity.

### 4.7. Recellularization

Human-induced pluripotent stem cells-derived MSCs were used for seeding, or the so-called recellularization of the decellularized valved stent. This process started with the incubation of the decellularized valved stent with MSC growth medium (consisting of Dulbecco’s modified Eagle’s medium-high glucose (DMEM-HG) (Thermo Fisher Scientific), 10% FBS, and 1% PS) in a humidified incubator at 37 °C and 5% CO_2_ overnight. The next day, and again on the following day, the MSC cells were extracted from the culture flasks (~8 × 10^6^ cells each) and added directly onto the valve surface. The seeding process was conducted on a cell seeder (Aptus Bioreactors, Clemson, SC, USA) in the incubator, starting with 3 h of rest to facilitate cell adhesion onto the leaflet surface, and continuing with the motion program of the seeder (2 rpm at a 30° angle to each side for 1 h, followed by 30 min rest, in repetition). To ensure an equal distribution of all the cells onto each of the three leaflets, the stent was rotated regularly during the process. The culture medium was refreshed two times per week. After 21 days, the leaflets were separated from the stent and were washed carefully in warm PBS. Finally, they were fixed in phosphate-buffered formaldehyde (ROTI*Histofix 10%, Roth) for 4 h at 4 °C for further analysis.

### 4.8. Biomechanical Test

The biomechanical characteristics of the native and decellularized leaflets were analyzed by uniaxial tensile testing. Tensile tests were performed using a universal testing machine (ZwickRoell Z0.5, Ulm, Germany) with a 0.5 kN load cell with a sensitivity of 2 mV/V. The samples were stretched in both the radial and circumferential directions at 5.0 mm/min until complete rupture. Young’s modulus, F_max_, and elongation at break were recorded for these measurements as described before by [19]. 

### 4.9. Heart Valve Test

**Figure 8:**
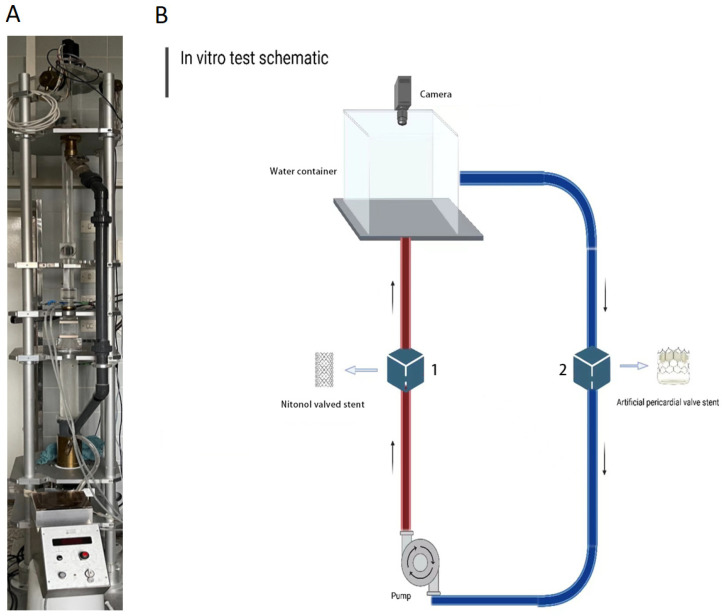
The test system simulates the cardiac cycle (**A**) and the operating principle of the test system (**B**).

The valve stent testing system was utilized to simulate the cardiac cycle, by conducting mechanical tests and morphological observations on the valved stents (Figure 8A). The test system includes a pump for establishing fluid circulation; two containers, one for the valved stent and the other for the countered prosthetic pericardial valve; a camera used to observe the valve opening and closing status; and pressure transducers for systolic and diastolic pressure monitoring. The pressure above and below the valve was measured for both systolic and diastolic, and then the systolic and diastolic transvalvular gradient could be measured. Figure 8B shows the operating principle of our test system. The opening area and pressure data for the valve were collected. After the completion of the testing, a gross macroscopic analysis was conducted.

### 4.10. Statistical Analysis

The statistical software R, version 4.3.0 [43] was used to evaluate the data. For the DNA quantification, the data evaluation started with the definition of an appropriate statistical model based on the generalized least squares [44], for both the aortic and pulmonary valves. The influence factor treatment had three levels: the native porcine control (NP), the positive control (PC), and the newly developed protocol, the experimental treatment (ET). The residuals were assumed to be normally distributed and to be heteroscedastic. These assumptions were based on a graphical residual analysis [45]. Based on this model, the relative efficiency of the ET to PC was tested according to Hasler et al. [46]. The corresponding results were presented as simultaneous, one-sided 95% confidence intervals (instead of *p*-values).

For the biomechanical test data, appropriate linear models were used for both the radial and circumferential measurement variables. Two influence factors with an interaction effect were considered: the treatment (with levels NP and ET) and type (pulmonary, aortic). The residuals were assumed to be normally distributed and to be homoscedastic. Based on this model, equivalence tests [47,48] were conducted for the treatments ET and NP. The corresponding results were presented as simultaneous, two-sided 90% confidence intervals (instead of *p*-values).

## Figures and Tables

**Table 1:** The mechanical test variables (E_mod_, F_max_, elongation at F_max_, and UTS) are presented as the mean ± SD. Further, the differences between the mean values of the ET and NP, along with their corresponding two-sided 90% confidence intervals (CI 90), are shown. The parameters that are significantly equal for the ET and NP are filled in gray. The ET and NP are accepted as significantly equivalent when the CI 90 ranges are located within technically acceptable limits.

**E_mod_ (MPa)**	**Radial**				**Circumferential**			
	**Aortic**		**Pulmonary**		**Aortic**		**Pulmonary**	
	**ET**	**NP**	**ET**	**NP**	**ET**	**NP**	**ET**	**NP**
Mean ± SD	13.05 ± 5.71	9.98 ± 5.01	6.64 ± 3.95	7.02 ± 4.33	64.22 ± 43.44	168.65 ± 25.43	40.46 ± 22.98	66.26 ± 23.63
Difference	3.06		−0.38		−104.43		−25.81	
CI 90	(−2.53; 8.66)		(−4.88; 4.12)		(−137.68; −71.18)		(−59.72; 8.10)	
**F_max_ (N)**	**Radial**				**Circumferential**			
	**Aortic**		**Pulmonary**		**Aortic**		**Pulmonary**	
	**ET**	**NP**	**ET**	**NP**	**ET**	**NP**	**ET**	**NP**
Mean ± SD	2.62 ± 0.99	2.28 ± 0.58	1.44 ± 0.45	1.30 ± 0.29	11.09 ± 6.59	20.78 ± 1.87	6.50 ± 3.28	14.28 ± 2.16
Difference	0.33		0.13		−9.69		−7.78	
CI 90	(−0.39; 1.06)		(−0.45; 0.72)		(−14.28; −5.10)		(−12.46; −3.10)	
**Elongation at F_max_** **(%)**	**Radial**				**Circumferential**			
	**Aortic**		**Pulmonary**		**Aortic**		**Pulmonary**	
	**ET**	**NP**	**ET**	**NP**	**ET**	**NP**	**ET**	**NP**
Mean ± SD	60.30 ± 17.61	61.74 ± 27.06	65.81 ± 28.21	77.23 ± 25.29	45.48 ± 7.91	41.79 ± 6.52	39.75 ± 11.68	48.38 ± 14.09
Difference	−1.44		−11.42		3.69		− 8.64	
CI 90	(−31.28; 28.40)		(−35.45; 12.60)		(−6.95; 14.33)		(−19.49; 2.22)	
**UTS (MPa)**	**Radial**				**Circumferential**			
	**Aortic**		**Pulmonary**		**Aortic**		**Pulmonary**	
	**ET**	**NP**	**ET**	**NP**	**ET**	**NP**	**ET**	**NP**
Mean ± SD	5.24 ± 1.99	4.57 ± 1.16	2.88 ± 0.89	2.61 ± 0.58	22.19 ± 13.18	41.57 ± 3.74	13.00 ± 6.56	28.57 ± 4.32
Difference	0.67		0.27		−19.38		−15.96	
CI 90	(−0.78; 2.11)		(−0.89; 1.43)		(−28.56; −10.20)		(−24.92; −6.20)	

**Table 2:** The systolic and diastolic pressure (mmHg) gradients are presented as the mean ± SD. Further, the differences between the mean values of the ET and NP, along with their corresponding two-sided 90% confidence intervals (CI 90) are shown. The parameters that are significantly equal in the ET and NP are filled in gray. The ET and NP are accepted as significantly equivalent when the CI 90 ranges are located within technically acceptable limits.

Pressure Gradient (mmHg)	Systolic				Diastolic			
	Aortic		Pulmonary		Aortic		Pulmonary	
	ET	NP	ET	NP	ET	NP	ET	NP
Mean ± SD	2.58 ± 0.55	4.99 ±1.92	2.40 ± 0.51	3.83 ± 1.18	10.89 ± 3.31	10.65 ± 2.88	11.85 ± 1.85	7.96 ± 3.62
Difference	−2.41		−1.42		0.24		3.89	
CI 90	(−4.3; −0.53)		(−3.46; 0.61)		(−4.48; 4.96)		(−1.21; 8.99)	

**Table 3:** The valve opening areas (mm^2^) at 60 bpm are presented as the mean ± SD. Further, the differences between the mean values of the ET and NP, along with their corresponding two-sided 90% confidence intervals (CI 90), are shown. The parameters that are significantly equal in the ET and NP are filled in gray. The ET and NP are accepted as significantly equivalent when the CI 90 ranges are located within technically acceptable limits.

Opening Area (mm^2^)	At 60 bpm			
	Aortic		Pulmonary	
	ET	NP	ET	NP
Mean ± SD	115.24 ± 37.13	62.84 ± 18.48	104.61 ± 18.1	92.40 ± 17.92
Difference	52.4		12.21	
CI 90	(15.41; 89.38)		(−24.77; 49.20)	

## Data Availability

The data that support the findings of this study are available in the Supplementary Materials for this article. The raw datasets for this study are available from the first author or corresponding author upon reasonable request.

## Supplementary Materials

The supporting information can be downloaded at: https://www.mdpi.com/article/10.3390/ijms25074026/s1.
