# Supplementary material for: Optimization of Enzymatic and Chemical Decellularization of Native Porcine Heart Valves for the Generation of Decellularized Xenografts
Source: Int J Mol Sci. 2024 Apr 4;25(7):4026. doi: 10.3390/ijms25074026 (PMC11012489; doi:10.3390/ijms25074026)
Supplement: Supplementary file 1 [file ijms-25-04026-s001.zip › ijms-2917985-supplementary.pdf]

S1. Supplementary Materials

S1.1. Visual appearance

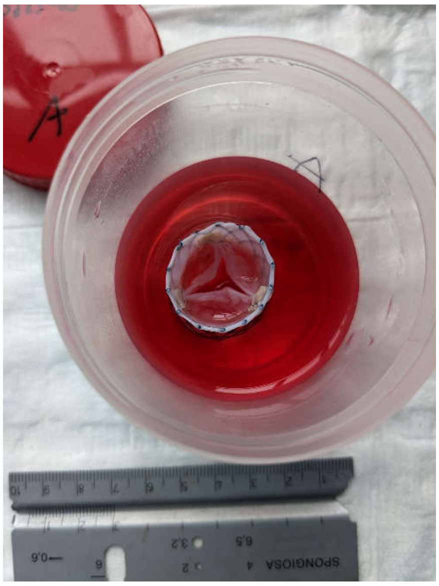

Figure S1. Represents the original image that was used to adjust the image for section C in Figure 1.

S1.2. DNA quantification

In a pilot study, 23 different protocols (Table S1) were studied. The residual DNA after decellularization by each protocol was analyzed and compared (Figure S2). Since protocols 5 and 16 were only tested with aortic leaflets and protocol 17 was exclusively conducted with pulmonary leaflets, these protocols were excluded from the pilot study. Besides, in protocol 5, even though the DNA concentration remained very low, the leaflets appeared fragile and viscid.

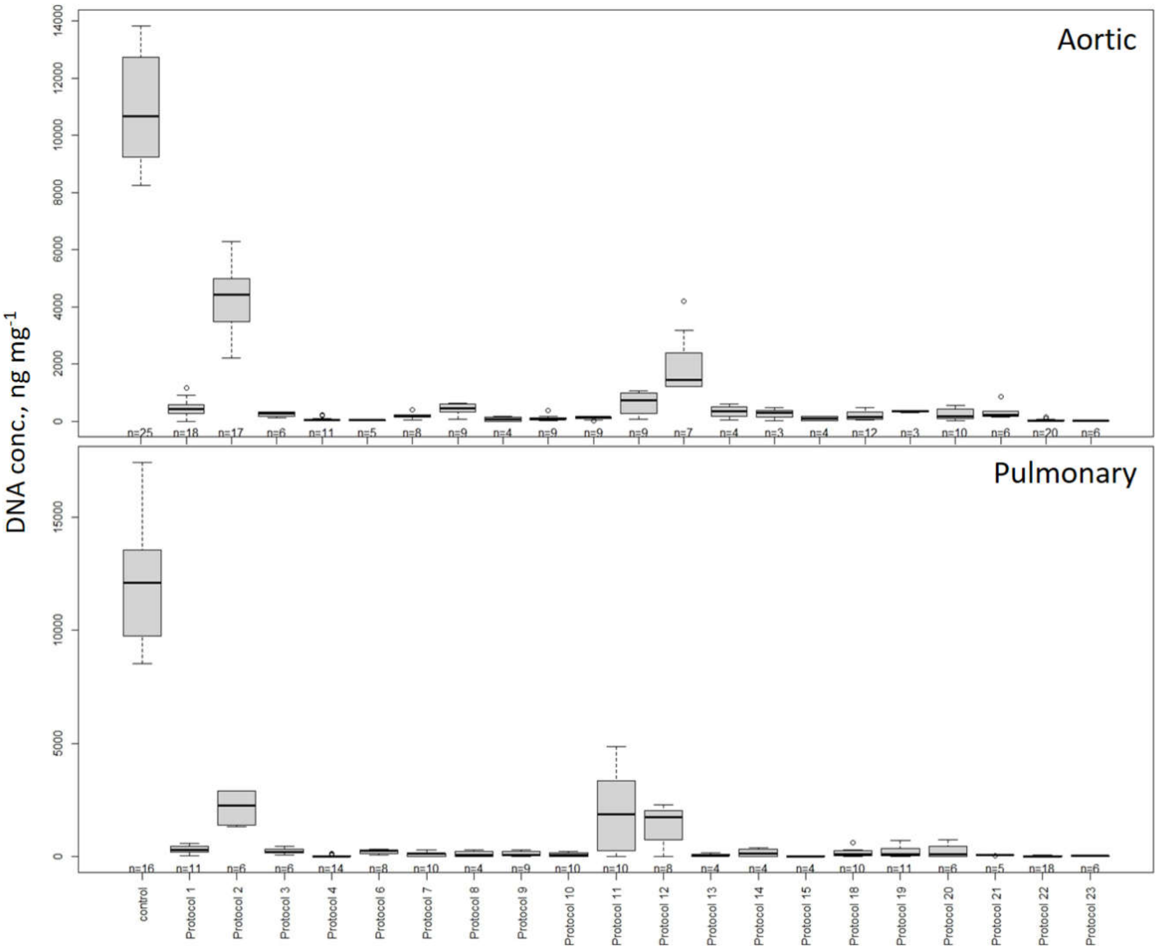

Figure S2. Absolute (boxplots) quantification of DNA content of untreated native and decellularized aortic and pulmonary valves by different protocols in the pilot study. Differences in the protocols are highlighted in Table S1.

	Tergitol	Triton X-100, 1%	Trypsin (g)	DCA (g)	DNase (Units)	RNase (Units)	SDS (g)	PMSF	NaN3 0.02%	0.05M NaOH, (hour)	CaCl2 (mmol)	70% EtOH (min)	NaCl (g)	Duration (day)
Native leaflets (NP)	-	-	-	-	-	-	-	-	-	-	-	-	-	-
Protocol 1 (PC)	-	✓	-	1	72	72	0.2	-	-	2	-	20	-	10
Protocol 2	-	✓	0.05	-	2500	2500	-	✓	✓	1	-	-	-	6
Protocol 3	-	✓	0.05	-	2500	2500	-	✓	✓	1	1	30	-	6
Protocol 4	-	✓	0.05	-	2500	2500	-	✓	✓	1	2	30	-	6
Protocol 5	-	✓	0.05	0.2	2500	2500	-	✓	✓	1	2	30	-	6
Protocol 6	-	✓	0.05	-	2500	2500	-	✓	✓	1	2	30	-	5
Protocol 7	-	✓	0.05	-	1250	1250	-	✓	✓	1	2	30	-	5
Protocol 8	-	✓	0.05	-	1875	1875	-	✓	✓	1	2	30	-	5
Protocol 9	-	✓	0.05	-	625	625	-	✓	✓	1	2	30	-	5
Protocol 10	-	✓	0.05	-	312.5	312.5	-	✓	✓	1	2	30	-	5
Protocol 11	-	✓	0.0	-	156.25	156.25	-	✓	✓	1	2	30	-	5
Protocol 12	-	✓	0.05	-	78.125	78.125	-	✓	✓	1	2	30	-	5
Protocol 13	✓	-	0.05	-	312.5	312.5	-	✓	✓	1	2	30	-	5
Protocol 14	-	✓	0.035	-	312.5	312.5	-	✓	✓	1	2	30	-	5
Protocol 15		-	0.05	-	312.5	312.5	-	✓	✓	1	2	30	-	5
Protocol 16	-	✓	0.05	-	625	625	-	✓	✓	1	2	30	-	5
Protocol 17	-	✓	0.05	-	312.5	312.5	-	✓	✓	1	2	30	-	5
Protocol 18	✓	-	0.035	-	312.5	312.5	-	✓	✓	1	2	30	-	5
Protocol 19	-	✓	0.035	-	312.5	312.5	-	✓	✓	1	2	30	-	5
Protocol 20	-	✓	0.035	-	625	625	-	✓	✓	1	2	30	-	5
Protocol 21	✓	-	0.035	-	625	625	-	✓	✓	1	2	30	-	5
Protocol 22 (ET)	✓	-	0.035	-	625	625	-	✓	✓	1	2	30	1.17	3 ½
Protocol 23	-	✓	0.035	-	833	833	-	✓	✓	1	2	30	1.17	3 ½

Table 1S. The table highlights the main differences. The concentrations refer to the usage for 100 ml. In this table '-' and '✓' mean 'not used' and 'used', respectively.

S1.3. Histology and immunohistochemistry analysis; original images

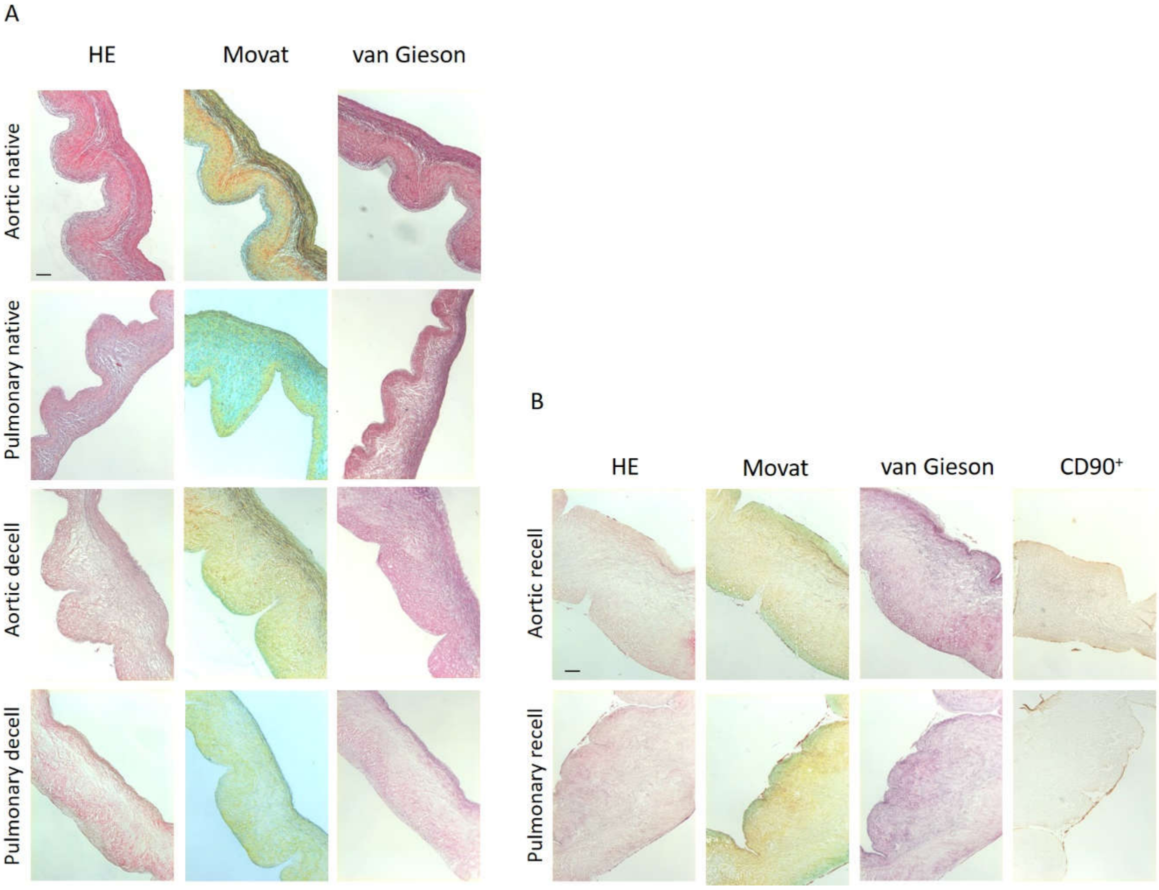

Figure S3. Represents the original images that were used to prepare Figure 4. in the manuscript, images of aortic and pulmonary leaflet tissues before decellularization (native) and after decellularization (decell) (A), and after recellularization (recell) (B), and stained with HE, Movat, van Gieson, and CD90+. The scale bar indicates 100 µm (A,B).

Author Contribution

Conceptualization, Jette Seiler, Georg Lutter, Thomas Pühler and Janarthanan Sathananthan; Funding acquisition, Georg Lutter; Investigation, Lena Floder, Jette Seiler and Monireh Saeid Nia; Methodology, Lena Floder, Jette Seiler, Nina Pommert, Rouven Berndt, David Meier, Zhang Xiling, Mario Hasler and Monireh Saeid Nia; Resources, Stanislav N. Gorb; Software, Zhang Xiling and Mario Hasler; Supervision, Jette Seiler, Georg Lutter, Stephanie Sellers, Gregor Warnecke and Monireh Saeid Nia; Validation, Jette Seiler; Writing – original draft, Lena Floder, and Monireh Saeid Nia; Writing – review & editing, Lena Floder, Jette Seiler, Georg Lutter, Thomas Pühler, Nina Pommert, Rouven Berndt, David Meier, Stephanie Sellers, Janarthanan Sathananthan, Zhang Xiling, Mario Hasler, Gregor Warnecke and Monireh Saeid Nia.

Funding

Financial support was received from the German Centre for Cardiovascular Research (DZHK).

Acknowledgements

The authors would like to greatly thank our former colleague, Philipp Knüppel (current address: Römer Engineering, Germany, https://roemer-engineering.de/) for his support in measuring and analyzing the mechanical properties as well as morphological results of the valved stent.

Conflicts of Interest

The authors declare no conflict of interest.
